# The antihypertension drug doxazosin suppresses JAK/STATs phosphorylation and enhances the effects of IFN-α/γ-induced apoptosis

**DOI:** 10.18632/genesandcancer.37

**Published:** 2014-11

**Authors:** Mi Sun Park, Boh-Ram Kim, Sokbom Kang, Dae-Yong Kim, Seung Bae Rho

**Affiliations:** ^1^ Research Institute, National Cancer Center, Ilsandong-gu, Goyang-si Gyeonggi-do, Republic of Korea; ^2^ Department of Veterinary Pathology, College of Veterinary Medicine, Seoul National University, Gwanak-gu, Seoul, Republic of Korea

**Keywords:** doxazosin, interferon-α/γ, apoptotic cell death, JAK/STAT activation, cell cycle progression

## Abstract

Doxazosin, a commonly prescribed treatment for patients with benign prostatic hyperplasia, serves as an α1-blocker of the adrenergic receptors. In this study, we calculated its effect on the ovarian carcinoma cells. Doxazosin induces dose-dependent growth suppression and is additively activated through IFN-α or IFN-γ stimulation. They both enhanced G1 phase arrest, as well as the activity of caspase-3, and the reduction of cyclin D1 and CDK4 protein levels. Doxazosin growth suppression was abolished either by the Janus family of tyrosine kinase (JAK) or the signal transducer and activator of transcription (STAT) inhibitor treatment. The activity of JAK/STAT was dependent on the level of doxazosin, suggesting a requirement of doxazosin for the activation of JAK/STAT. Furthermore, doxazosin plus IFN-α or doxazosin plus IFN-γ additively suppressed the activation of the JAK/STAT signals through phosphorylation of JAK and STAT, thus affecting the activation of subsequent downstream signaling components PI3K, mTOR, 70S6K, and PKCδ. *In vivo* study demonstrated that doxazosin significantly suppressed tumor growth in an ovarian cancer cell xenograft mouse model, inducing apoptotic cell death by up-regulating the expression of p53, whereas c-Myc expression was markedly reduced. Our data indicate that doxazosin can modulate the apoptotic effects of IFN-α- and IFN-γ through the JAK/STAT signaling pathways. Collectively, we indicate that this action may be a potent chemotherapeutic property against ovarian carcinoma.

## INTRODUCTION

Doxazosin {1-(4-amino-6,7-dimethoxy-2-quinazolinyl)-4-(1,4-benzodioxan-2-ylcarbonyl) piperazine methanesulfonate}, quinazoline compound, is known as an α1-blocker of adrenergic receptors [1]. Generally, α1-blockers have similar abilities to β-blockers during biological functions, which they function to block α-receptors in the blood and heart physiological system, such as relaxing blood vessels and reducing the speed and force of the heart's contractions [2].

Interferons (IFNs) are a glycoprotein known as being part of the cytokine superfamily, and are known to regulate the response of the immune system, such as during invasions from viruses, parasites, bacteria, and cancer cells. IFNs directly control the immune system response and decrease the growth of carcinoma cells by mediating the action of various types of genes [3, 4]. In addition, IFNs regulate the phosphorylation of STAT by interacting with their specific receptor. IFN-α is directly developed from leukocytes and are mostly involved in both anti-viral and immunoregulatory activities at target cells. IFN-α is also a key indicator for viral infections through Toll-like receptors (TLRs), where macrophages and natural killer (NK) cells are induced, which enhances antigen presentation to lymphocytes [5-7]. IFN-γ is a secreted protein that serves as a regulator of macrophage activation, where it appertains to the type II class of the interferon family. It is also activated by specific antigen presentation and can induce apoptosis. In addition, IFN-γ can induce the anti-tumor effect, anti-viral, and immunoregulatory of the type I interferons, such as IFN-α, IFN-β, IFN-ω, IFN-κ, and IFN-ε [8-10]. Previous studies have demonstrated that IFN-γ can either direct anti-proliferative activity or induce apoptotic cell death in some ovarian carcinoma cell types, as well as in primary carcinoma cells and mouse tumor model systems [4, 11-13].

The JAK/STAT pathways are involved in the regulation of the immune system for various cellular events including cell proliferation, differentiation, hematopoiesis, development, and apoptosis. They are activated by the binding of cell-surface receptors, such as the bindings by interferons, growth factors, and interleukins [14, 15]. Interrupted or non-regulated JAK/STAT functionality can result in various cancers and immune disorders. For instance, STAT3 is continuously up-regulated in various tumors, including major carcinomas and some hematologic cancers, while JAK activation induces cell proliferation, cell migration, and apoptotic cell death [16].

Doxazosin is also prescribed to patients with benign prostatic hyperplasia, a non-cancerous enlargement of the prostate gland, because α1-blockers alleviate the smooth muscles surrounding the prostate, easing urine flow and decreasing bladder outlet obstruction [17, 18]. These drugs can also cause erectile dysfunction, although not as frequently as some other blood pressure medications. In some studies, α1-adrenoceptor antagonists have induced apoptotic cell death through the TGF-β1 signaling IκBα activation in prostate carcinoma cells [17, 19]. Doxazosin has also been validated to induce apoptotic cell death, in which the antagonistic effector is triggered by abnormal cell-matrix interactions [20]. In a recent study, doxazosin-mediated apoptosis could be blocked by specific caspase-8 inhibitors in benign prostate cells (BPH). Caspase-8 activation is initiated by its interaction with FADD and their subsequent recruitment by doxazosin [21]. However, the details of the molecular mechanism of these doxazosin-mediating processes are still poorly understood.

This study was designed to explore the major roles of the critical components in the cellular signaling pathways, such as JAK/STAT phosphorylation, accompanied by the patterns of expression of apoptosis-related proteins by doxazosin-induced apoptosis along with the underlying molecular mechanisms of ovarian carcinoma cells.

## RESULTS

### Doxazosin inhibits cell proliferation in a dose-dependent manner, and additively enhances apoptotic cell death by IFN-α and IFN-γ treatment

**Figure 1:**
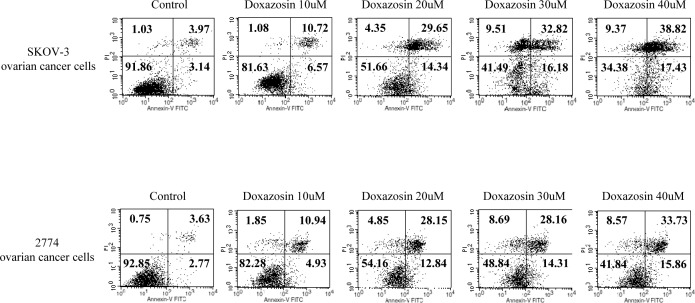
Effects of doxazosin on cell proliferation in SKOV-3 and 2774 ovarian carcinoma cells Exponentially-growing cells were treated with increased concentrations of doxazosin (0-40 μM). The cell death distribution calculated for the presence of apoptotic cells using flow cytometry with a fluorescein isothiocyanate (FITC)-labeled Annexin V assay.

**Figure 2:**
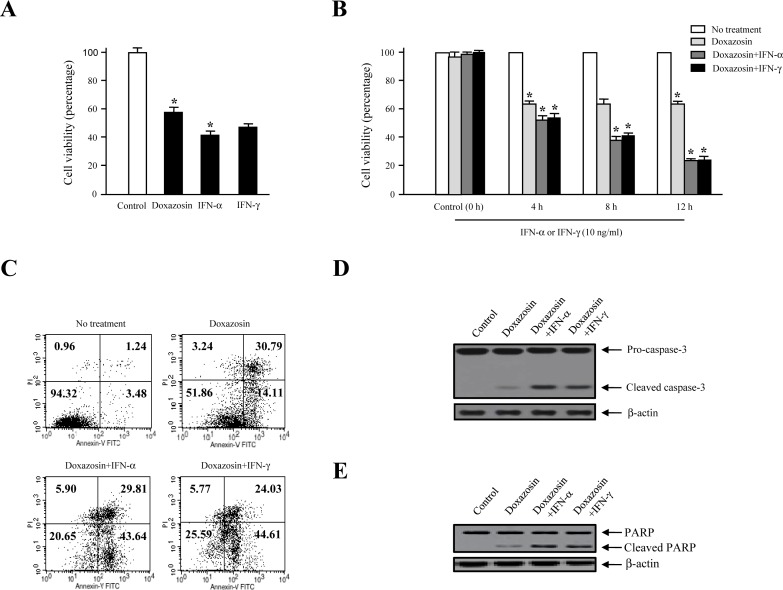
Additive effects of doxazosin by IFN-α or IFN-γ stimulation (A) SKOV-3 cells were treated with doxazosin, IFN-α, or IFN-γ and the cell viability was measured using a MTT colorimetric assay. (B) IFN-α and IFN-γ-stimulated cells were treated for the indicated times with doxazosin. Cell proliferation was evaluated using 3-(4,5-dimethylthiazol-2-yl)-2.5-diphenyl-2H-tetrazolium bromide (MTT) reduction. Significant differences of 95% confidence (*P*<0.05) are depicted with an asterisk (*) for each graph. (C) Cells were treated with control (untreated) or doxazosin alone or in combination (doxazosin plus IFN-α and doxazosin plus IFN-γ), where the data calculated the additive effects of doxazosin in combination using the flow cytometry system. (D-E), Caspase-3 and PARP cleavages activated by doxazosin, doxazosin plus IFN-α, or doxazosin plus IFN-γ treatments. Soluble protein extracts were introduced by immunoblotting for cleaved caspase-3 and cleaved PARP. β-actin was used to verify equal loading.

To study the biological function of doxazosin during IFN-α< or IFN-γ treatment in carcinoma cells, we elected to use a treatment of doxasozin which is known as an α1-blocker of adrenergic receptors. The cell viability of SKOV-3 cells treated with various concentrations (0, 10, 20, 30, and 40 μM) of the doxazosin drug were decreased in a dose-dependent manner, where the doses were required to suppress the growth of the cells by 40%, which exceeded 20 μM (Fig. 1, upper panel). Similar results of the pro-apoptotic effect were shown by other ovarian carcinoma cells, such as 2774 (lower panel) and OVCAR-3 (data not shown). We then examined the apoptotic activity of doxazosin through IFN-α and IFN-γ treatment, respectively. As presented in Fig. 2A, the effect of each doxazosin, IFN-α, and IFN-γ on SKOV-3 cells, decreased the number of cells, which is indicative of cell viability. IFN-α treatment significantly reduced the proliferation in the cells. The cell viability of IFN-α-treated SKOV-3 cells was inhibited to approximately 60% when compared to the untreated cells. In contrast, doxazosin and IFN-γ weakly inhibited the cells. We also calculated the potential additive or synergistic effects of doxazosin in combination with IFN-α or IFN-γ in ovarian tumors. As shown in Fig. 2B, co-treatment with doxazosin and IFN-γ or IFN-α, had an additive effect, indicating that doxazosin plus either, IFN-α or IFN-γ, additively inhibits cell growth in a time-dependent manner. The cytotoxicity of the doxazosin/IFN-α or IFN-γ combination on carcinoma cells were evaluated using fluorescence activated cell sorting (FACS) with the FITC-labeled Annexin V. As presented in Fig. 2C, pre-treatment with IFN-α or IFN-γ dramatically promoted doxazosin-induced apoptotic cell death, showing that IFN-α or IFN-γ enhances doxazosin-mediated cell growth inhibition. Caspases can be activated through intrinsic or extrinsic signaling pathways. Caspase-3 is a critical apoptotic molecule, as it is responsible for the proteolytic cleavage of various regulatory proteins, such as poly (ADP-ribose) polymerase (PARP). To confirm the increased activity of the cleaved caspase-3, the immunoblotting of major pro-caspase-3 and cleaved caspase-3 involved in the apoptotic pathways is presented in Fig. 2D. The immunoblotting results that were clearly observed dramatically enhance the activity of caspase-3 with doxazosin alone, doxazosin plus IFN-α, or doxazosin plus IFN-γ treatment. Subsequently, the cleavage of PARP induced by doxazosin alone was also additively induced by the combination with each IFN-α and IFN-γ treatment (Fig. 2E).

### Effect of doxazosin on IFN-α- or IFN-γ-stimulated expression of cell cycle modulator proteins

**Figure 3:**
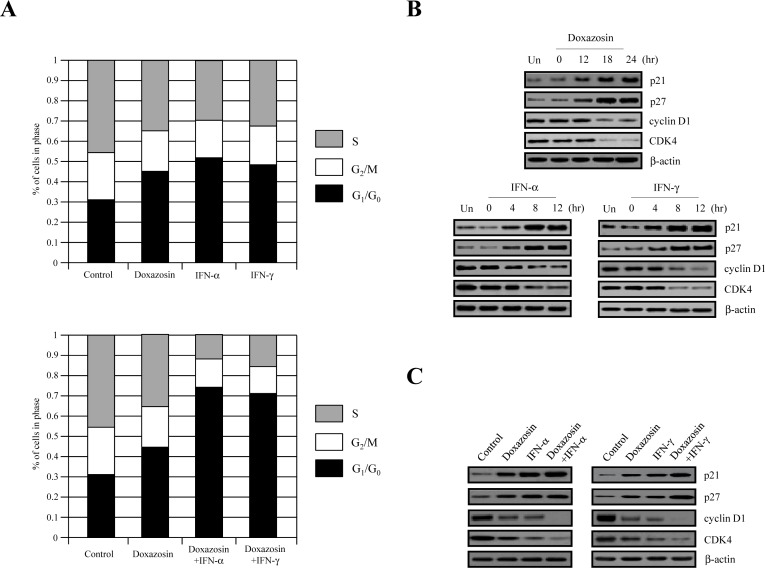
Effects of doxazosin on IFN-α or IFN-γ-stimulated cell cycle distribution and cell cycle-associated protein expression (A) Cells were pre-treated with IFN-α or IFN-γ, then with doxazosin, collected, and analyzed using flow cytometry. The cell cycle distribution was assessed form the presence of apoptotic cells using FITC-annexin V and PI. (B-C) Effects of doxazosin on the expression levels of IFN-α or IFN-γ-stimulated cell cycle-associated proteins. Cells were treated to compare with a time-course of shorter times (0, 12, 18, 24 h) by using the indicated control (untreated cells), doxazosin alone, IFN-α alone, IFN-γ alone, or combination. Cell lysates were immunoblotted with the indicated antibodies (p21, p27, cyclin D1, and CDK4). β-actin was used as a loading control.

To investigate whether cell cycle arrest is related to the expression of the cell cycle regulatory proteins, as well as changes to the cell cycle distribution, cells were treated at various times with the indicated doxazosin, IFN-α, IFN-γ, or combination. As presented in Fig. 3A (upper panel), the cell cycle profile of the control (untreatment) cells did not change significantly, except that of the G_1_ phase section gradually decreasing due to doxazosin, IFN-α, or IFN-γ treatment, respectively. In addition, the cell cycle profile of the cells treated with the doxazosin plus IFN-α or doxazosin plus IFN-γ combinations arrested the S phase when compared to the doxazosin single-drug treatment (lower panel). Interestingly, the greatest pro-apoptotic effect occurred with the combination of 20 μM of doxazosin and 10 ng of IFN-α. These results suggest that the induction of apoptotic cell death is contributed by the doxazosin plus IFN-α-mediated anti-tumor effect. The expression of cyclin D1 and CDK4, which are relevant to the conversion from the G_1_ to S phase, was inhibited in a time-dependent manner. In addition, cyclin-dependent kinase (CDK) inhibitors p21 and p27, which typically cause cells to delay cell cycle progression in the G1 phase, were induced in the doxazosin, IFN-α, or IFN-γ-treated cells (Fig. 3B). Consistent with these findings, a decrease in cyclin D1 and CDK4 protein levels in response to the combined treatment was greater than that of doxazosin, IFN-α, or IFN-γ-treated alone cells (Fig. 3C). Taken together, our findings indicate that doxazosin, IFN-α, and IFN-γ inhibit cell proliferation by enhancing G_1_ phase arrest in ovarian carcinoma cells.

### Doxazosin inhibits phosphorylation of JAK/STAT

**Figure 4:**
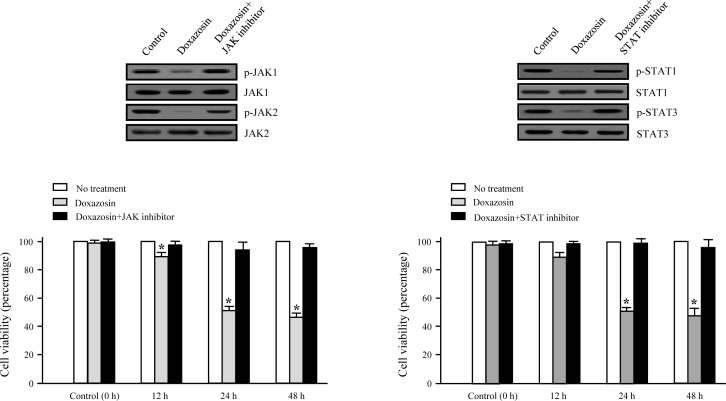
JAK/STAT inhibitors block doxazosin-mediated JAK/STAT dephosphorylation Cells were treated with control (untreated cells), doxazosin, doxazosin plus JAK1/2 inhibitor, or doxazosin plus STAT1/3, respectively. Cell lysates were blotted with the indicated antibodies (upper panel). Levels of JAK1 (total and p-Y1022/1023), JAK2 (total and p-Y1007/1008), STAT1 (p-Y701), and STAT3 (total and p-Y705) were developed by Western blotting. Cells were treated for the indicated times with the indicated drug agents (lower panel). Experiments were performed in triplicate and error bars are shown as the mean±SD. *P<0.05.

Considering the propagation of JAK/STAT activation in malignant cancer and the requirement of the various biological/physiological responses for cell proliferation, understanding how JAK/STAT activation regulates the phosphorylation levels and cellular signal transduction processes is very important. JAK proteins are phosphorylated when cytokines bind to their specific receptors, subsequently activating STATs. STATs are a family of cytoplasmic proteins with Src homology-2 (SH2) domains that act as signal messengers and transcription factors [22]. Based on the findings presented, we examined whether doxazosin suppressed the phosphorylation of JAK and STATs in carcinoma cells. As presented in Fig.4, phosphorylation of JAK and STAT were dramatically reduced by doxazosin. In addition, JAK and STAT inhibitor treatment exactly recovered doxazosin-reduced phosphorylation of both JAK1/2 and STAT1/3. Consistent with the results observed in carcinoma cells viability, inhibitors for JAK and STAT also significantly blocked doxazosin-reduced JAK or STAT activity (Fig. 4). Taken together, these results suggest new insight into the anti-tumor effects of this potentially important new chemotherapeutic agent in ovarian carcinoma.

### Decreased phosphorylation of PI3K/Akt/mTOR as well as 70S6K and PKCδ following doxazosin treatment

**Figure 5:**
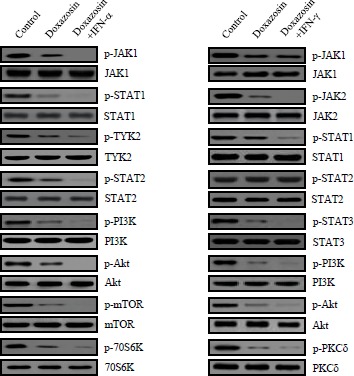
Doxazosin down-regulates the phosphorylation of the major components of the JAK/STAT/mTOR-mediated signaling pathways Cells were treated with control (untreated cell), doxazosin alone, doxazosin plus IFN-α, or doxazosin plus IFN-γ. Then, the cell proteins, JAK1, JAK2, Tyk2, STAT1, STAT2, STAT3, PI3K, Akt, mTOR, 70S6K, and PKCδ were phosphorylated, and were developed through immunoblotting with specific antibodies for each activated proteins (p-JAK1, p-JAK2, p-Tyk2, p-STAT1, p-STAT2, p-STAT3, p-PI3K, p-Akt, p-mTOR, p-70S6K, p-PKCδ). Western blots for the non-phosphorylated proteins were used as a loading control (JAK1, JAK2, Tyk2, STAT1, STAT2, STAT3, PI3K, Akt, mTOR, 70S6K, PKCδ).

IFNs are crucial regulators of the JAK/STAT signaling pathway. The IFN-α/β receptors consist of two subunits of IFNR-1 and IFNR-2. Generally, activation of the JAK family (JAK1 and TYK2) followed by the phosphorylation of the STAT1/2 proteins. In contrast, IFN-γ leads to the phosphorylation of the JAK1/2 tyrosine kinases, resulting in the phosphorylation of STAT1 through the binding of its receptor. As with previous evidence, immunoblotting was employed to evaluate the effect of doxazosin on the JAK/STAT signaling pathway molecules, such as Tyr2, PI3K, Akt, mTOR, 70S6K, and PKCδ phosphorylation. Generally, active PI3K/Akt induces cell proliferation as well as protein synthesis through the activation of the mTOR and 70S6K genes. Cells were treated with 20 μM doxazocin, 20 μM doxazoxin plus 10 ng IFN-α, or 20 μM doxazosin plus 10 ng IFN-γ, respectively. As presented in Fig. 5, doxazoxin down-regulates phospho-JAK1/2 and STAT1/3, but not the total JAK1/2 and STAT1/3. Consistent with this, doxazosin plus IFN-α or IFN-γ additively inhibited the activation of the JAK/STAT pathway, and thus affected subsequent downstream signaling components, including PI3K, mTOR, 70S6K, and PKCδ activation. Overall the results validated that doxazosin can potentiate the effects of IFN-α or IFN-γ through JAK/STAT/mTOR signaling pathway-dependent apoptotic cell death, as well as increasing G_1_ phase arrest of the cell cycle progression.

### Doxazosin inhibits tumor growth in nude mice

**Figure 6:**
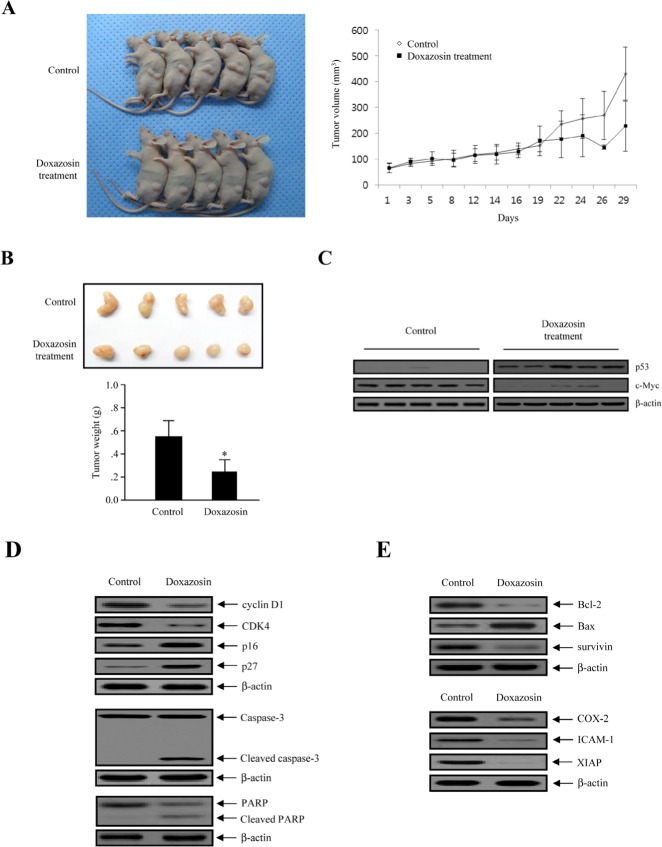
Doxazosin suppresses tumor progression in nude mice (A) Tumor growth an ovarian carcinoma xenograft model. Subcutaneous tumors derived from the SKOV-3 cells were treated with doxazosin as described. Tumor volumes are evaluated as the mean of at least five mice per group. *P<0.05 compared to the control. (B) After sacrificing, image of the xenograft tumor mass treated with or without doxazosin (upper panel). Inhibitory effects of doxazosin on xenograft tumor weight (lower panel). * indicates *P*<0.05 (compared to control). (C) Effect of doxazosin treatment on the expression level of p53 in SKOV-3 carcinoma cell-derived tumors excised on the 31st day post-treatment, measured using Western blot analysis. (D) From xenografted mice, soluble protein extracts were detected by immunoblot for the indicated proteins (cyclin D1, CDK4, p16, p27, caspase-3 and PARP). β-actin was used as a loading control. Un-cleaved caspase-3 and PARP and their cleaved products are indicated. (E) Protein expression of anti-apoptotic/oncogenic/anti-proliferation genes (Bcl-2, Bax, survivin, COX-2, ICAM-1 and XIAP) were developed using Western blot. β-actin protein was used as an equal loading control.

We investigated the growth-inhibitory effect of doxazosin on ovarian carcinoma cells *in vivo*. For this purpose, Exponentially-growing SKOV-3 ovarian cells were implanted subcutaneously into immune-deficient BALB/c nude mice. We allowed the tumors to grow until they reached a mean volume of 50 mm3. Tumor growth was inhibited by doxazosin at the 20th day, which reached a mean volume of 100 mm3 (Fig. 6A). There were no differences in body weight loss or liver toxicity (data not shown). The volume of the treated tumors was 50-65% smaller than those of the control mice (Fig. 6B). Next, we examined the effect of doxazosin on p53 stabilization in tumor tissues collected from the control and doxazosin treated mice. Doxazosin treatment was observed to remarkably increase the level of p53 protein expression, whereas c-Myc expression was markedly reduced (Fig. 6C). Subsequently, to further clarify the biological mechanism of doxazosin-induced cell cycle arrest, the protein levels of cell progression were examined with tumor tissue lysates. The expression levels of cyclin D1 and cyclin dependent kinase 4 (CDK4) protein, which is associated with the transition of G_1_ to S phase, was significantly reduced, whereas the CDK cell cycle inhibitors p16 and p27, which are related with the interruption of cell cycle procession in the G or G_2_ /M phase, were enhanced (Fig. 6D, upper panel). In addition, to address whether doxazosin induces apoptotic cell death, we evaluated the activation of caspase-3 for stimulation of apoptosis, as well as the inactivation of poly (ADP-ribose) polymerase (PARP) through Western blot analysis. Expression of cell death-related proteins such as caspase-3 and PARP was remarkably increased in doxazosin-treated tumors (Fig. 6D, lower panel). These results indicate that doxazosin is capable of inhibiting tumor growth by inducing apoptotic cell death *in vivo*. We next showed that Bax expression was increased in the doxazosin-treated group, but not in the control group (Fig. 6E, upper panel). Meanwhile, the protein expression of anti-apoptotic/oncogenic/anti-proliferation genes, such as Bcl-2, survivin, cyclooxygenase (COX-2), intercellular adhesion molecule 1 (ICAM-1) and X-linked inhibitor of apoptosis protein (XIAP) were decreased in the doxazosin treatment group, compared to the control group (Fig. 6E). Collectively, these results showed that the treatment with doxazosin significantly suppressed tumor growth in the mouse ovarian cancer cell xenograft model.

## DISCUSSION

Our studies were planned to analyze the mechanism of the effects of doxazosin on the JAK/STAT signaling pathway by IFN-α or IFN-γ stimulation in ovarian carcinoma cells. This was due to the fact that the JAK/STAT pathways are a rising interest for targeting cancer-associated diseases, which also include cancer treatment. In the biological function, type I IFN-α and IFN-β are multifunctional cytokines, as major modulators of the innate and adaptive immune system that signal through the JAK/STAT pathway in the activation of STAT1/2 [23]. In case of a response to IFN-α, which is preceded by IFN-α binding to cell surface receptors, JAK kinases (TYK2 and JAK1) are activated, leading to tyrosine phosphorylation of STAT1 and STAT2. Generally, cytokines and their cognate transmembrane receptors are the essential activators of the JAK/STAT pathway. IFNs regulate both anti-viral and immunomodulatory activities, while mediating intracellular effects, such as the anti-tumor and anti-angiogenic effects. Recently, we revealed direct evidence that DAPk1 is a major regulator of IFN-γ-induced apoptotic cell death in human ovarian carcinoma cells [24]. In this study, our results strongly indicate a new crucial molecular mechanism for doxazosin, which can be reported as a key regulatory factor that can target the JAK/STAT signaling cascade. As shown in Fig. 2C, pre-treatment with IFN-α or IFN-γ significantly increased doxazosin-induced apoptotic cell death, which was observed through IFN-α or IFN-γ up-regulating doxazosin-modulated cell growth inhibition. Furthermore, the cleavage of PARP, which can be activated by doxazosin alone, was also additively promoted via the combinations with IFN-α and IFN-γ treatment (Fig. 2E).

Activation of STATs can be mediated through IFN-α, IFN-β, or IFN-γ signaling, where it is subsequently translocated to the nucleus. IFN-α/β directly bind to their receptors and trigger auto-phosphorylation of the JAK protein families, such as JAK1, JAK2, and TYK2. Previous studies have shown that STAT1 is involved in mechanisms like cell proliferation and apoptotic cell death, so phosphorylation-independent STAT1 biological functions have been postulated [25, 26]. Meyer et al. [27] have reported that STAT1 can be found in both the cytoplasm and the nucleus without cytokine induction of cells. Also, cytomegaloviral proteins demonstrated a dual biological function for STAT2 in anti-viral responses and IFN-γ signaling [28]. In addition, Dengue virus NS5 suppresses interferon-α signaling through the blocking of STAT2 phosphorylation [29]. Other recent studies have revealed STAT2 as a key component of the STAT1-independent mechanism for the protection against DENV infection in mice, which has validated that STAT1 and STAT2 possess the ability to independently limit the severity of DENV pathogenesis [30]. Interestingly, STAT3 up-regulates various proteins involved in cell cycle progression, such as Fos, c-Myc, and cyclin-D, and enhances anti-apoptotic proteins, such as Bcl-2 and Bcl-xL [31]. Thus, STAT3 target proteins are important mediators for the regulation of cell cycle progression from the G1 to S phase.

The effects of doxazosin on the biological roles of the IFN-α or IFN-γ cascade in ovarian tumors have not been fully understood. To explain the biological mechanism of cell proliferation suppression by doxazosin concerning cell cycle changes, we observed both, an inhibition of cell cycle-regulatory proteins such as cyclin D1 and CDK4, and expression of p21 in doxazosin-treated carcinoma cells. As indicated in Fig. 3A (upper panel), the cell cycle profile of the control (un-treatment) cells were not affected significantly, except at the S phase section which was gradually down-regulated by doxazosin, IFN-α, or IFN-γ treatment. Additionally, the cell cycle profile of the cells treated with doxazosin plus IFN-α or doxazosin plus IFN-γ combinations, were arrested at the S phase when compared to the doxazosin single-drug treatment (lower panel). Interestingly, the greatest pro-apoptotic effect occurred with the combination of 20 μM of doxazosin and 10 ng of IFN-α. These results indicate that the activation of apoptosis is contributed by the doxazosin plus IFN-α combination-modulated anti-tumor effect. The expression of cyclin D1 and CDK4, which are relevant for the conversion from the G1 to S phase, was suppressed in a time-dependent manner. The present study also demonstrated that doxazosin significantly down-regulates the phosphorylation of JAK/STAT in SKOV-3 cells, which can work in combination with the JAK and STAT inhibitors. JAK and STAT inhibitor treatment completely restored the doxazosin-reduced phosphorylation of each JAK1/2 and STAT1/3. Consistent with the results observed in the ovarian carcinoma cells, inhibitors for JAK and STAT also strongly disturbed doxazosin-reduced JAK or STAT activity (Fig. 4B). Subsequently, doxazosin plus IFN-α or IFN-γ additively suppressed the activation of the JAK/STAT pathway, and thus affecting the subsequent downstream signaling components, which include PI3K, mTOR, 70S6K, and PKCδ activation.

In conclusion, we have shown that doxazosin can significantly inhibit the JAK/STAT signaling pathway through caspase-dependent apoptosis, as well as cell cycle arrest by IFN-α or IFN-γ stimulation. Growth suppression was associated in the ovarian tumor system with the down-regulation of the JAK/STAT activity that resulted in down-stream signaling suppression. These findings provide the first exact information on the biological pro-apoptotic mechanisms of doxazosin in carcinoma cells. Therefore, combination therapies may be a more useful approach for more advanced ovarian cancers and recurrent patients.

## MATERIALS AND METHODS

### Cell lines, drug reagents, and antibodies

Human ovarian cancer cell lines (SKOV-3, 2774 and OVCAR-3) were obtained from the American Type Culture Collection (ATCC, Manassas, VA). Human IFN-α and IFN-γ were obtained from Sigma (St. Louis, MO). The chemical doxazosin was also purchased from Sigma. The JAK1/2 kinase inhibitor INCB18424 (Ruxolitinib) and STAT1 inhibitor (NSC118218) were obtained from Selleck Chemicals (Houston, TX), and stock solutions were prepared in DMSO. NSC74859 (S31-201), a specific STAT3 inhibitor, was purchased from Calbiochem Chemicals (La Jolla, CA). The following primary antibodies were used in this study: anti-JAK1, anti-phospho-JAK1, anti-JAK2, anti-phospho-JAK2, anti-STAT1, anti-phospho-STAT1, anti-STAT3, anti-phospho-STAT3, anti-caspase-3, anti-cMyc, anti-Bcl-2, anti-Bax, anti-p53, anti-survivin, and anti-COX-2 (Cell Signaling, Beverly, MA), anti-PARP, anti-XIAP (BD Biosciences, San Jose, CA), anti-cyclin D1, anti-CDK4, anti-Akt, anti-phospho-Akt, anti-TYK2, anti-phospho-TYK2, anti-PI3K, anti-phospho-PI3K, anti-mTOR, anti-phospho-mTOR, anti-PKCδ, anti-phospho-PKCδ, anti-STAT2, anti-phospho-STAT2, anti-p70S6K, and anti-phospho-p70S6K (Santa Cruz Biotechnology, Santa Cruz, CA), anti-p21, and anti-p27 (Oncogene, San Diego, CA).

### Cytotoxicity assay

Cell viability was evaluated using 3-(4,5-dimethylthiazol-2-yl)-2.5-diphenyl-2H-tetrazolium bromide (MTT) assays. Briefly, cells were seeded at 5.5×103 cells per well in 96-well plates. Then, the cells were treated with doxazosin, IFN-α, or IFN-γ and 20 μl of MTT solution (Sigma, 5 mg/ml) were added to each well. The plate was incubated for an additional 4 h at 37°C. The MTT reaction was terminated with DMSO.

### Cell cycle analysis

Ovarian carcinoma cells were plated onto chamber slides at a density of 4.5×104 cells per well and were then treated. A cell cycle distribution was performed through fluorescence-activated cell sorting (FACS). Cells were harvested by trypsinization and rinsed twice with phosphate buffered saline (PBS). After centrifugation, cells were incubated with FITC-labeled Annexin V and propidium iodide (PI) for 15 min according to the manufacturer's protocols (BD PharMingen, Mississauga, ON).

### Western blotting

Cells and tissues were collected, rinsed twice with cold PBS, and lysed using RIPA lysis buffer with protease inhibitor cocktail (Sigma) at 4°C for 50 min. Cell lysates containing equal amounts of protein were subsequently separated with 8-12% SDS-PAGE, where they were then transferred onto Hybond-ECL nitrocellulose membrane (GE Healthcare, UK). After blocking, the membranes were incubated with the appropriate primary antibodies at 4°C overnight. The membranes were washed thrice with TBST buffer and incubated in either goat anti-rabbit or anti-mouse secondary antibodies.

### Xenograft mouse model

Specific pathogen-free BALB/c - nu/nu mice (5-6 week old) were purchased from Orientbio (Sungnam, Korea). All animal studies were approved by the Institutional Animal Care and Use Committee (IACUC) at the Research Institute of the National Cancer Center. To establish ovarian tumors in mice, 1.0×106 SKOV-3 cells were injected s.c. with Matrigel (BD Bioscience, MA) into the mid-dorsal region of each nude mouse. Tumors were allowed to grow for 10 days. Tumor sizes were measured by caliper measurements 3 times every week. On day 10, oral treatment of 3 mg/kg of doxazosin was done and repeated 5 times per week for 30 days. Mice were sacrificed, one day after the final treatment, and tumors were then excised and stored at −80°C for further study.

### Statistical analysis

Data are expressed as the mean±SD of error for the mean of the triplicate experiments. Statistical analyses were performed using Student's t-test for comparisons between the two groups. The index for statistical significance was *P*<0.05. The values with 95% confidence (*P*<0.05) are depicted with an asterisk (*) on each graph.

## Abbreviations

IFN-α/γ: interferon-alpha/gamma
JAK: Janus family of tyrosine kinase
STAT: signal transducer and activator of transcription
TLRs: toll-like receptors
PARP: poly (ADP-ribose) polymerase
SH2: Src homology-2.
